# Microscopic Epididymal Sperm Aspiration (MESA) Should be Employed Over Testicular Sperm Extraction (TESE) Sperm Retrieval Surgery for Obstructive Azoospermia (OA)

**DOI:** 10.7759/cureus.40659

**Published:** 2023-06-19

**Authors:** Hatsuki Hibi, Megumi Sonohara, Miho Sugie, Noritaka Fukunaga, Yoshimasa Asada

**Affiliations:** 1 Urology, Kyoritsu General Hospital, Nagoya, JPN; 2 Medical Coordinator, Asada Ladies Clinic, Nagoya, JPN; 3 Embryologist, Asada Institute for Reproductive Medicine, Nagoya, JPN; 4 Obstetrics and Gynecology, Asada Institute for Reproductive Medicine, Nagoya, JPN

**Keywords:** sperm retrieval surgery, assisted reproductive technology (art), intracytoplasmic sperm injection (icsi), testicular sperm extraction (tese), microscopic epididymal sperm aspiration (mesa), obstructive azoospermia (oa)

## Abstract

Introduction: Testicular sperm extraction (TESE) has been widely used as a sperm extraction surgery for azoospermia even for obstructive azoospermia (OA) because it does not require surgical skill. However, there are postoperative pain issues, and subsequent testicular atrophy and decreased testosterone levels may occur with TESE. This study examines the usefulness of microscopic epididymal sperm aspiration (MESA) for OA.

Methods: We studied 108 patients diagnosed with OA and treated with MESA at our institute between April 2004 and December 2021. The MESA was performed using a micropipette with a micropuncture technique under an operative microscope. When no sperm were present or motility was not observed, additional punctures to the epididymal tubule were performed.

Results: Motile sperm were recovered in all cases (108 cases). Of these, intracytoplasmic sperm injection (ICSI) using frozen-thawed sperm was performed in 101 cases and the normal fertilization rate was 76.2%. A total of 436 embryo transfer (ET) cycles were performed. The implantation rate per transfer cycle was 47.9%, the clinical pregnancy rate was 41.0%, and the live birth rate was 23.7%. The per-case live birth rate was 84.8%.

Conclusions: MESA-ICSI has a very good fertilization rate, clinical pregnancy rate, and delivery rate. Furthermore, the patient's postoperative pain is less, the number of sperm collected is larger, the burden on the embryologist who processes the collected sperm is less, and ICSI can be easily attempted after frozen-thawed sperm. MESA rather than TESE should be employed for the OA subjects.

## Introduction

Testicular sperm extraction (TESE) is technically simple, and micro-TESE is widely used as a sperm extraction technique because it does not require microsurgical skills. However, since the testicle is incised, the pain, which is a symptom of peritoneal irritation, is severe. Furthermore, there have been reported postoperative testicular atrophy and decreased testosterone levels with TESE. The processing of TESE specimens is complicated and has had a significant impact on the workload of laboratories, which has been increasing in recent years. Although the testicular incision is theoretically unnecessary in obstructive azoospermia (OA), TESE is widely used as a sperm collection method even in OA because of its technical simplicity as described above.

Since 1992, we have been using microscopic epididymal sperm aspiration (MESA) for OA. However, it has been argued that when MESA specimens fail to achieve assisted reproductive technology (ART) results, it is not the fertilization process but the sperm that is the problem. We retrospectively evaluated 108 patients diagnosed with OA treated by MESA at our institute. These data were previously presented at the 60th Annual Meeting of the Japanese Society for Fertilization and Implantation held in Tokyo.

## Materials and methods

Study design, size, and duration

One hundred and eight patients diagnosed with OA and treated with MESA at the Asada Ladies Clinic between April 2004 and December 2021 were included in this report. We examined the results of ART using sperm collected by MESA.

Patients and setting

Diagnosis of OA was made by repeated semen analyses, careful palpation, ultrasound (US), hormonal test, and chromosomal testing. OA is considered to be defined as normal testicular volume, gonadotropins, and chromosomes. The causes of obstruction were the congenital bilateral absence of vas deferens (CBAVD) in four cases, unilateral epididymal and vas deferens defects in two cases, failed vasovasostomy in two cases, tuberculous epididymitis, vasectomy, and after posterior urethral valve surgery in one case, respectively, and other causes were unknown.

MESA

Microscopic epididymal sperm aspiration was carried out by using a micropuncture technique under local anesthesia with a spermatic block [1]. The preparation of the micropipette used for micropuncture was previously described in an animal experimental setting [2]. Micropipettes were made by drawing on a horizontal puller from constant-bore flint glass tubing with a diameter of 1 mm and an inside diameter of 0.7 mm. The pipette tips were sharpened on a rotating wet stone grinder to a diameter of about 75 μm to facilitate penetration of the epididymal tubule wall. The epididymal tunic capsule was minimally incised with microscissors, and the epididymal tubule was directly punctured by a micropipette under a microscope with 15× to 20× magnification. Epididymal fluid was gently aspirated. In principle, the puncture site was the caput of the epididymis. If no sperm were present or no motile sperm could be recovered, micropuncture was repeatedly performed at a different site. The reason is that it is possible to obtain motile sperm by puncturing from the epididymal tubule, which is slightly distant from the initial puncture site. Aspirated samples were examined and transferred into modified human tubal fluid and sent to the in vitro fertilization (IVF) laboratory for cryopreservation.

Sperm counting, freezing, and thawing

The collected sperm were counted under a microscope. If sperm were not frozen, they were used for insemination after counting. Sperm freezing was performed as follows: sperm was washed several times with 4-(2-hydroxyethyl)-1-piperazineethanesulfonic acid (HEPES) medium (Sperm Washing Medium, SW012, Nakamedical Inc., Japan) and centrifuged at 300g for 10 min. After centrifugation, the supernatant was removed, leaving the pelleted sperm, and resuspended in sperm freezing solution (Freezing Medium, 90128, FUJIFILM IrvineScientific, Inc., CA). Sperm freezing straws (Sperm Freeze Straw 0.5 mL, NFA101, Nakamedical Inc., Japan) were filled with PBS, air, sperm solution, air, and phosphate-buffered saline (PBS) in this order, and the ends of the straws were closed with straw powder (Straw Powder, FA349, Nakamedical Inc., Tokyo, Japan), and after standing for 10 min, the sperms were placed in liquid nitrogen for cryopreservation.

The following procedure was used to thaw the sperm. Straws containing sperm solution were removed from liquid nitrogen, allowed to stand at room temperature for 30 s, and then placed in hot water at 30°C-35°C for 1 min. The straws were then cut with scissors and the sperms were collected in 15-mL tubes; HEPES medium was added to the sperm solution, mixed lightly, and centrifuged at 300g for 10 min. After centrifugation, the supernatant was removed, leaving the sperm in pellet form. The sperm pellets were resuspended in pentoxifylline solution adjusted to 3.6 mM in HEPES medium and used for insemination.

Protocol for controlled ovarian stimulation [3]

Human menopausal gonadotropin (Ferring Pharmaceuticals, Tokyo, Japan or ASKA Pharmaceutical, Tokyo, Japan) or recombinant FSH (Gonal-f®; Merck Serono, Tokyo, Japan) was used for controlled ovarian stimulation (COS). In the long protocol, buserelin 600 μg/day (Buserecur®; Fuji Pharma, Tokyo, Japan) was administered intranasally on day 21 of the prestimulation cycle, and in the short protocol, buserelin 600 μg/day was administered intranasally on day 3 of the stimulation cycle. In both protocols, a gonadotropin-releasing hormone (GnRH) agonist was administered daily until the day of human chorionic gonadotropin (hCG) injection. The GnRH antagonist protocol involved alternate-day administration of a GnRH antagonist [ganirelix (Ganirest®, MSD, Tokyo, Japan) or cetrorelix (Cetrotide®, Merck Serono)] when follicular size reached a diameter of 14-16 mm or when a premature luteinizing hormone (LH) surge was suspected based on serum LH levels, a GnRH agonist or hCG (3000-5000 IU) was administered for final oocyte maturation. Mature oocytes were retrieved transvaginally after 34-36 h of hCG/GnRH agonist injection, depending on follicular diameter, serum estradiol (E2) and anti-Müllerian hormone (AMH) levels, and the patient's age. The retrieved oocytes were then fertilized by intracytoplasmic sperm injection. Fresh embryo transfer was performed if the following criteria were met: (a) the serum E2 level on the day of hCG injection was ≤6000 pg/mL, (b) the serum progesterone (P4) level on the day of hCG injection was ≤1.5 ng/mL, and (c) no signs of ovarian hyperstimulation syndrome (OHSS) were observed after oocyte retrieval.

Embryo culture and cryopreservation

Embryos were cultured individually from day 3 to day 5 for fresh embryo transfers, while for freeze-thaw embryo transfer, embryos were frozen at the pronuclear or blastocyst stage using either slow freezing or vitrification methods. Slow freezing was accomplished using the Embryo Freezing Pack and Embryo Thawing Pack (Origio, Måløv, Denmark), while the vitrification method used the Vitrification Kits VT101 and VT102 (Kitazato BioPharma, Shizuoka, Japan).

Embryo transfer [4]

Frozen-thawed embryo transfers were conducted in artificial hormone replacement cycles following the endometrial preparation protocol with a combination of transdermal E2 (Estrana®, Hisamitsu, Saga, Japan) and chlormadinone acetate (Lutoral®, FujiPharma, Tokyo, Japan). The E2 treatment began on the second or third day of the artificial hormone replacement cycle, and the endometrial thickness was measured from day 9 to day 11 of the cycle. If the endometrium thickness was ≥7 mm, the frozen-thawed embryo transfer was scheduled. Chlormadinone acetate treatment began on day 15 of the cycle with a dose of 6 mg/day. The cleavage-stage embryos were transferred on day 3, considering the starting day of chlormadinone acetate treatment as day 0. The blastocyst-stage embryos were transferred on day 6, considering the starting day of chlormadinone acetate treatment as day 0. For fresh embryo transfers, if pregnancy occurred, transdermal estradiol at a dose of 2.16 mg every two days and chlormadinone acetate at a dose of 12 mg/day was given. For both fresh and frozen-thawed embryo transfers, transdermal E2 at a dose of 2.16 mg every two days and chlormadinone acetate at a dose of 6 mg/day were administered until nine weeks of gestation. Pregnancy was confirmed by urinary hCG measurement and transvaginal ultrasound scan. When the urinary hCG level on day 14 after embryo transfer was ≥50 IU/mL, the pregnancy test was regarded as positive, and the presence of an intrauterine gestational sac was verified one week later via a transvaginal ultrasound scan.

Institutional Review Board

The protocol for this research project, including its use of human subjects, was approved by a suitably constituted Asada Ladies Clinic Ethical Committee. Our approval number is 2020-18, and the date of approval is September 30, 2020.

## Results

Median patient age was 35 (interquartile range; 30, 39) and the median duration of infertility was 2 years (1, 3). The median testicular volume was 20 mL (16, 24) on the right and 18 mL (15, 22) on the left. The median hormonal panel of LH, follicle-stimulating hormone (FSH), and testosterone were 3.85 mIU/mL (2.51, 5.07), 4.24 mIU/mL (3.25, 5.90), 5.04 ng/mL (3.70, 6.03), respectively. Median body mass index (BMI) was 23.2 (21.4, 24.7) kg/m2. Motile sperm were recovered from all 108 men who underwent MESA between 2004 and 2021. Aspirated epididymal sperm concentrations were shown in Table 1. Of these cases, 101 underwent IVF cycles (Table 2). The median age of the female partners at the time of oocyte retrieval was 32 (interquartile range; 29, 37). The median anti-Müllerian hormone (AMH), LH, FSH, and BMI were 2.82 ng/mL (1.35, 4.90), 5.30 mIU/mL (4.35, 6.45), 7.15 mIU/mL (6.48, 8.30), and 20.5 kg/m2 (18.8, 22.7), respectively. Among 245 IVF cycles, fresh sperm was used for insemination in five (2%) cycles and thawed sperm in 240 (98%) cycles. A total of 2530 MII oocytes were recovered in 245 cycles and inseminated by intracytoplasmic sperm injection (ICSI), resulting in 1929 fertilized oocytes with two pronuclei, for an overall fertilization rate of 76.2%. A total of 436 transfer cycles were performed after MESA-ICSI, with 67 fresh embryo transfers (15.4%) and 369 frozen embryo transfers (84.6%). Of these, the pregnancy outcome was unknown for five fresh embryo transfers and 26 frozen-thawed transfers (pregnancy outcome was unknown for nine cases). The implantation rate per transfer cycle was 47.9%, the clinical pregnancy rate was 41.0%, and the live birth rate was 23.7%. The per-case live birth rate was 84.8%.

**Table 1:** Aspirated epididymal sperm concentration.

Aspirated sperm	n=108
Less than 1 x 10^6^/mL, n (%)	2 (1.9)
1 x 10^6^ to 10 x 10^6^/mL, n (%)	4 (3.7)
More than 10 x 10^6^/mL, n (%)	102 (94.4%)

**Table 2:** Results of ART. Median (interquartile range); n (%) ART, assisted reproductive technology; IVF, in vitro fertilization; OPU, oocyte pick up; ET, embryo transfer †Number excluding cycles with unknown pregnancy outcome ‡Number excluding patients with unknown pregnancy outcome

Characteristic	n=101
Spouse age (years)	32 (29, 37)
Number of IVF cases performed	101
Number of OPU cycles	245
Spermatozoa used for insemination (cycle)	
Fresh, n (%)	5 (2.0)
Freeze-thaw, n (%)	240 (98.0)
Total number of mature oocytes	2530
Median number of mature oocytes	7 (3, 17)
Total number of two-pronuclear embryos	1929
Median number of two-pronuclear embryos	5 (2, 11)
Two-pronuclear fertilization rate, n (%)	1929/2530 (76.2)
Embryo transfer cycles	436
Fresh embryo transfer, n (%)	67 (15.4)
Freeze-thaw embryo transfer, n (%)	369 (84.6)
Median number of embryos transferred	1 (1, 2)
Implantation rate / ET cycle^†^, n (%)	194/405 (47.9)
Clinical pregnancy rate / ET cycle^†^, n (%)	166/405 (41.0)
Live birth rate / ET cycle^†^, n (%)	96/405 (23.7)
Live birth rate / patient^‡^, n (%)	78/92 (84.8)

## Discussion

Vasectomy, inflammation of the epididymis, inguinal hernia surgery congenital bilateral absence of vas deferens (CBAVD), etc. are thought to be the causes of OA; it is not treated in the same way as non-obstructive azoospermia (NOA). Initial therapy for these patients was microsurgical seminal reconstruction. However, this was not possible to complete in CBAVD or failed seminal reconstruction. Microsurgical vasoepididymostomy for unexplained epididymal obstruction is considered to be a challenging surgery due to the limited patency and natural pregnancy rates [5]. Sperm retrieval surgery requires that an adequate number of motile sperm can be retrieved with minimal damage to the patient. From this point of view, we believe that MESA should be employed for OA. Silber also reported that MESA was clearly associated with more mature spermatozoa than TESE, and testicular sperm consistently yielded lower results than epididymal sperm. He concluded that sperm origin, rather than spermatogenesis disorder, determines the success rate of ICSI [6].

Although many surgical methods are available for sperm retrieval, the choice of surgical method is based on the attending surgeon’s preference. Sperm extraction surgery is often performed by TESE. However, it is mandatory to consider the disadvantages to the patient and the burden on the embryologist who handles the sperm after retrieval surgery. From this point of view, we have been employing MESA for OA patients since 1992 [1]. 

In a systematic review and meta-analysis, the transient but statistically clear decrease in testosterone levels after TESE indicated that men are at risk of developing hypogonadism after TESE [7]. Postoperative decreased testosterone level in subjects with various types of azoospermia has been reported. Eliveld et al. recently showed that an obvious effect of TESE on testosterone over time was observed in OA subjects, but not in men with NOA [8]. In their study, TESE for OA was performed on only one testicle.

Some have argued that when using MESA specimens fails to achieve assisted reproductive technology (ART) results, it is not the fertilization process but due to a sperm problem. For example, it has been reported that since MESA specimens contain DNA-fragmented sperm when compared with specimens of TESE, the ICSI results in poorer fertilization and pregnancy rates [9-10]. On the other hand, one study of 368 ICSI cycles in 171 OA patients showed an obviously higher abortion rate when sperm extracted from a testicle was used [11]. Van Wely et al. reported that in the first ICSI cycles of couples with OA, the use of epididymal sperm resulted in a significantly higher live birth rate than did the use of testis sperm [12]. In a meta-analysis setting, Nicopoullos et al. noted that fertilization rates varied from 45% to 72% for epididymal sperm and from 34% to 81% for testicular sperm. They concluded that MESA should be employed in view of the possible complication of testicular damage [13].

Uncontaminated sperm such as blood can be retrieved only by MESA, and there is no need for special procedures before cryopreservation. Previously we reported that MESA completed using the micropuncture technique resulted in higher fertilization and pregnancy rates [14]. The advantage of MESA is that a large quantity of sperm can be cryopreserved in a single procedure for future attempts at ICSI and that a clinical pregnancy rate of 42%-60% can be achieved [15-16]. Furthermore, Hayon et al. recently reported that MESA had similar ART results as ejaculated sperm in patients with OA [17]. In our study, the two-pronuclear fertilization rate was 76.2%, and 436 embryo transfer cycles were performed. The implantation rate per transfer cycle was 47.9%, the clinical pregnancy rate was 41.0%, and the live birth rate was 23.7%. The per-case live birth rate was 84.8%. Thus, our data are enough comparable with those mentioned above other MESA-ICSI reports.

Collaboration with other professions is extremely important in medical care. An embryologist minces the tissue and uses a microscope to locate sperm. Testicular tissue mincing and subsequent finding sperm might be a laborious process depending on the degree of sperm production. On the other hand, although MESA requires microsurgical skill, it yields a high number of motile sperm. MESA can reduce the laboratory workload of embryologists which has been increasing in recent years because no tissue mincing is required and the cryopreservation process is straightforward.

Using the MESA procedure, even if the aspirated fluid was very limited, sperm is highly concentrated (approximately 1 x 106/μL) in the epididymal fluid [18]. We also previously reported that sufficient sperm could be retrieved in MESA specimens [1], and in this study, 92% of cases had a sperm concentration of 10 x 106/mL. It is easier to apply MESA specimens to ICSI when the sperm is fresh or frozen and thawed. Bernie et al. also reported that the advantage of epididymal sperm is that it is clean and easy to cryopreserve for use in the in vitro fertilization laboratory, and its higher numbers compared to testicular sperm may allow patients to avoid invasive procedures in the future [19]. It should be emphasized that since MESA does not involve incision of the testes, there are fewer postoperative peritoneal irritation symptoms and no concerns about postoperative testicular atrophy or low testosterone levels. Postoperative pain was mild, and most patients used it only once or twice usage of oral analgesics. And there is no need to worry about long-term effects on testosterone, unlike after TESE [8]. In OA patients, the disadvantages of incision of the testis to the patient should be fully considered before deciding on the surgical method. We had not been employing TESE as the method for sperm retrieval in OA patients. We believe that MESA should be preferred unless TESE-ICSI is clearly better than MESA-ICSI for OA patients.

Limitations, reasons for caution

Some authors have reported that since MESA specimens contain DNA-fragmented sperm when compared with specimens of TESE, the ICSI results in poorer fertilization and pregnancy rates. We have not evaluated sperm DNA fragmentation. With regard to sperm DNA fragmentation, the 2021 World Health Organization laboratory manual for examination states that it is not a standard test and evidence is still controversial.

Wider implications of the findings

A large quantity of uncontaminated sperm can be retrieved using only MESA with less invasive for the patient, and there is no need for special requirements before cryopreservation. It can reduce the laboratory workload of embryologists which has been increasing in recent years.

## Conclusions

The MESA-ICSI can result in a very good fertilization rate, clinical pregnancy rate, and delivery rate. MESA should be employed for the OA subjects, not TESE.
